# Remote intelligent perception system for multi-object detection

**DOI:** 10.3389/fnbot.2024.1398703

**Published:** 2024-05-20

**Authors:** Abdulwahab Alazeb, Bisma Riaz Chughtai, Naif Al Mudawi, Yahya AlQahtani, Mohammed Alonazi, Hanan Aljuaid, Ahmad Jalal, Hui Liu

**Affiliations:** ^1^Department of Computer Science, College of Computer Science and Information System, Najran University, Najran, Saudi Arabia; ^2^Department of Computer Science, Air University, Islamabad, Pakistan; ^3^Department of Computer Science, Applied College, King Khalid University, Abha, Saudi Arabia; ^4^Department of Information Systems, College of Computer Engineering and Sciences, Prince Sattam bin Abdulaziz University, Al-Kharj, Saudi Arabia; ^5^Computer Sciences Department, College of Computer and Information Sciences, Princess Nourah Bint Abdulrahman University (PNU), Riyadh, Saudi Arabia; ^6^Cognitive Systems Lab, University of Bremen, Bremen, Germany

**Keywords:** deep learning, remote sensing, image processing, intelligent perception, robotic environment, deep belief network, AlexNet

## Abstract

**Introduction:**

During the last few years, a heightened interest has been shown in classifying scene images depicting diverse robotic environments. The surge in interest can be attributed to significant improvements in visual sensor technology, which has enhanced image analysis capabilities.

**Methods:**

Advances in vision technology have a major impact on the areas of multiple object detection and scene understanding. These tasks are an integral part of a variety of technologies, including integrating scenes in augmented reality, facilitating robot navigation, enabling autonomous driving systems, and improving applications in tourist information. Despite significant strides in visual interpretation, numerous challenges persist, encompassing semantic understanding, occlusion, orientation, insufficient availability of labeled data, uneven illumination including shadows and lighting, variation in direction, and object size and changing background. To overcome these challenges, we proposed an innovative scene recognition framework, which proved to be highly effective and yielded remarkable results. First, we perform preprocessing using kernel convolution on scene data. Second, we perform semantic segmentation using UNet segmentation. Then, we extract features from these segmented data using discrete wavelet transform (DWT), Sobel and Laplacian, and textual (local binary pattern analysis). To recognize the object, we have used deep belief network and then find the object-to-object relation. Finally, AlexNet is used to assign the relevant labels to the scene based on recognized objects in the image.

**Results:**

The performance of the proposed system was validated using three standard datasets: PASCALVOC-12, Cityscapes, and Caltech 101. The accuracy attained on the PASCALVOC-12 dataset exceeds 96% while achieving a rate of 95.90% on the Cityscapes dataset.

**Discussion:**

Furthermore, the model demonstrates a commendable accuracy of 92.2% on the Caltech 101 dataset. This model showcases noteworthy advancements beyond the capabilities of current models.

## 1 Introduction

Scene recognition is a central field in the field of computer vision, where the goal is to use advanced computational techniques to break and classify complex visual robotic environments (Liu Y. et al., 2022; Liu D. et al., 2022; Liu H. et al., 2022; Wang et al., 2022). Understanding scenes and analyzing the object within the scene is a challenging task. It is a computational process that involves automated interpretation and categorization of visual information within the images. The process begins with extracting low-level features such as colors, textures, and shapes from the visual point. Subsequently, these features are utilized to construct higher level representations, enabling the system to recognize objects, spatial relationships, and contextual elements within the scenes. These systems have achieved the versatility of scene recognition, highlighting its significance across diverse fields such as smart home technologies (Zhou et al., 2020; Qi et al., 2022), surveillance systems (Sae-Ung et al., 2022), autonomous driving (Arnold et al., 2019), healthcare systems (Ulhaq et al., 2020; Angelica et al., 2021; Mehmood et al., 2022), and environmental monitoring (Yang B. et al., 2023; Yang D. et al., 2023).

For the previous two decades, researchers have been focusing on semantic segmentation, feature optimization, processing time, multi-object identification, and scene recognition. Object segmentation is presently used in various applications, including processing images, video identification, shadowing detection, human activity detection, and several others. It discussed techniques for static and moving object detection and segmentation but did not cover feature extraction techniques (Khurana et al., 2016). One of the most difficult problems in computer vision is semantic segmentation. The computer vision community is paying close attention to this task. A survey of RGB-D image semantic segmentation by deep learning may face limitations in the datasets, potential biases toward certain approaches, and challenges in addressing real-world variability and scalability (Noori, 2021). The method involves utilizing a pre-trained VGG16 model to extract features from input images and then using Random Forest for classification, displaying efficiency in image segmentation. This approach utilizes a pre-trained VGG16 model for feature extraction from input images, followed by classification using Random Forest. It has demonstrated effectiveness in image segmentation. However, its reliance on fixed, pre-defined CNN features restricts adaptability to diverse datasets and evolving model architectures. There are potential challenges in efficiently managing high-dimensional feature spaces (Faska et al., 2023). The article presents a comprehensive approach to scene recognition, comprising multiple sequential phases to ensure robust performance. It begins by ingesting raw data through various picture-acquisition methods, enabling the system to access diverse visual information. Subsequently, semantic segmentation techniques are applied to the data, enhancing its comprehension and usability by partitioning the scene into meaningful regions. This segmentation process facilitates the extraction of numerous object features, which are crucial for subsequent object recognition tasks employing deep belief models. Moreover, the system goes beyond individual object identification by analyzing object-to-object relationships, further enriching its understanding of composition and dynamics of the scene. Ultimately, scene recognition is accomplished through the utilization of an AlexNet neural network, leveraging its capabilities to discern complex patterns and configurations within the scene data. By adapting these phases in a systematic manner, the proposed system achieves a high level of resilience and efficacy in recognizing diverse scenes accurately. The primary findings and contributions of this study are outlined as follows:

- Utilizing UNet-based semantic segmentation, we segmented each object into homogeneous regions.
- We established a multi-feature strategy that included three separate sorts of features: Discrete Wavelet Transform, Sobel, Laplacian, and textual features.
- Object recognition was executed through the utilization of the deep belief network.
- The object-to-object relationship was found, followed by the AlexNet Neural Network for predicting scenes in the surroundings of scene recognition.

The sections of the article are organized as follows: Section 2 delves into a literature study on scene recognition. Section 3 discusses the suggested methodology in considerable detail. In Section 4. the experimental setup is delineated alongside the results obtained from conducted experiments, providing empirical insights into the system’s performance. Section 5 examines the system’s results and discusses its benefits and shortcomings. Section 6 is the conclusion, which summarizes the key findings and suggests future research and development objectives.

## 2 Literature review

There has been a tremendous surge in research activities in recent years, and efforts aimed at improving scene recognition systems, particularly in the context of both outdoor and interior situations. Contemporary research trends can be generally categorized into two major groups to draw linkages between the approaches suggested in this study and actual systems. These are semantic segmentation and scene recognition. The next sections expand on these areas, clarifying their contributions to the field’s research environment.

### 2.1 Multi-object segmentation

The research provides a semantic segmentation method for traffic image segmentation in the context of automated driving based on the UNET network architecture. By accurately segmenting traffic photos, the program attempts to increase the car’s understanding of the exterior scene. One limitation of this study is that the experiments were conducted using a specific dataset, the Highway Driving dataset. While this dataset is suitable for semantic segmentation tasks related to traffic scenes, the generalizability of the proposed algorithm to other datasets or real-world scenarios may need further investigation (Wang C. et al., 2023; Wang Q. et al., 2023). Shelhamer et al. (2017) convert existing classification networks into fully convolutional networks and employ a skip architecture to incorporate semantic and appearance information for accurate and thorough segmentation. Fully convolutional networks achieve enhanced segmentation on diverse datasets while keeping quick inference times. While they faced difficulty with gradient propagation when adding depth information to RGB images, challenges with gradient propagation can lead to issues such as vanishing or exploding gradients, hindering the network’s ability to learn effectively, difficulty in achieving fine-scale accuracy measured by mean IU metric, and high computational cost and complexity in using large filters for re-architecting layers. Class balancing methods have shown minimal improvement due to the slightly unbalanced nature of the labels. Liu D. et al. (2022), Liu Y. et al. (2022), and Liu H. et al. (2022) proposed that a CNN-based semantic segmentation is performed. It includes a Context Semantic Encoding (CSE) module for capturing global context information and emphasizing category information related to the scene. The generative confrontation network’s unsupervised data acquisition distribution rule is utilized to handle the spatial relationship between pixels, and a multi-scale extracted feature is employed to improve the value of the foreground targeted feature. The model struggle with capturing intricate spatial relationships between pixels due to the complexity of the scenes, potentially affecting segmentation accuracy. Badrinarayanan et al. (2017) utilized SegNet, a deep convolutional neural network framework, for semantic pixel-wise segmentation that comprises an encoding system, a decoding system, and a pixel-wise categorization layer. Compared with other architectures, it achieves efficient memory use and computational time during inference while giving good performance and competitive inference time. The author mentioned that the segmentation task faced challenges due to the large number of classes, especially smaller and less frequent ones, resulting in lower accuracy for these classes. Deep learning architectures such as VGG may struggle with indoor scene variability, with smaller models showing better performance. To address these issues, more comprehensive datasets and specialized training methods are needed for improved performance across varying class sizes and scene complexities. Rafique et al. (2022) demonstrated a convolutional neural net (CNN)-based segmentation method to recognize objects. CNN features are then obtained from these segmented objects, and discrete cosine transform and discrete wavelet transform features are computed. This fusion is achieved using fusion techniques after extracting CNN features and computing customary machine learning functions. Then, a minimal feature collection is selected using genetic algorithm-based feature selection. This study shows the great results but it did not mention the scene recognition accuracy results in terms of confusion matrix (Rafique et al., 2020). The proposed recognition technique is one kinf of a segmentation framework that uses probabilistic multi-object segmentation to train an accurate scene structure and separate objects in the scene. The distinguishing features of these segregated items are then obtained for further recognizing processing using linear SVM. Finally, the scene recognition features and weights are delivered to the multilayer perceptron. The proposed model’s performance may vary in complex real-world scenarios due to the limitations of depth data in capturing intricate scene details. The use of limited feature extraction techniques may impact scene recognition accuracy. Employing a variety of different features can enhance accuracy in scene recognition tasks.

Moreover, Herranz-Perdiguero et al. (2018) proposed that using semantic segmentation as input, the research provides a bottom-up strategy for solving the challenges of image pixel labeling, object recognition, and scene categorization. The ResNet deep network-based DeepLab architecture is used to accomplish precise pixel labeling, object localization, and scene identification. This model directly implements segmentation and detection techniques. By preprocessing images and extracting important details, it aims to achieve better results in scene analysis and recognition. Kim and Choi (2019) used a method for learning a new class containing backdrop and object for semantic image segmentation of inside scene photographs. The emphasis is on differentiating objects and backgrounds rather than learning different object classes, resulting in improved accuracy and less learning time. When the same class works independently across various environments, the suggested learning approach achieves approximately 5–12% higher accuracy than previous methods and lowers learning time by roughly 14–60 min. This method shows promise in quickly tackling the challenge of distinguishing objects and backgrounds in indoor photographs. This model operates solely on indoor scenes using a single dataset, which restricts its scalability and generalizability to broader contexts or outdoor environments. Das et al. (2019) achieved semantic segregation at the superpixel threshold employing three distinct levels as semantic context relatives. In addition, we used various ensemble techniques, such as maximum scoring and balanced mean. They also employed the Dempster–Shafer uncertainty theory to investigate class confusion. On the same dataset, our method outperformed a number of alternative recent approaches. The authors mentioned that they avoided incorporating higher combinations of classes because they would unnecessarily increase computational complexity without providing significant additional information. Specifically, when determining the predicted class of a patch, we excluded classes that were likely to be confused with the chosen class, reducing complexity while maintaining accuracy (Yoshihara et al., 2022). The study investigates the effect of training convolutional neural networks (CNNs) on ImageNet object recognition using a combination of sharp and blurry images. It finds that mixed training on sharp and blurred images makes CNNs closer to humans in terms of robust object recognition against changes in image blur, but it does not fully achieve a strong shape bias comparable to humans. The drawback of this approach is that training with blurred images did not noticeably enhance the recognition of overall spatial shapes or the use of fine details (high-frequency features) in object recognition tasks. Additionally, the models were trained on blurred images struggled to effectively use restricted frequency features and were particularly sensitive to local obstructions, which differs from how human vision handles similar challenges. Girshick et al. (2014) suggested an affordable and flexible detection technique that enhances the mean average precision (mAP) by more than 30% and achieves mAP of 53.3% in comparison to the prior highest results in VOC 2012. Two important insights are as follows: (1) Powerful convolutional neural networks (CNNs) can be used to find and segment objects from the ground-up area predictions; (2) When tagged training data are unavailable, pre-supervised data can be used to considerably increase performance through auxiliary task training and subsequent domain-specific fine-tuning. In region classification, accurately locating boundaries between different semantic regions in images can be challenging Wang et al. (2024), especially when objects overlap or are closely positioned. This can lead to less precise segmentation results as the method may struggle to assign accurate semantic labels to distinct regions.

### 2.2 Scene understanding

Scene understanding is a significant area in computer vision that aims to enable machines to perceive, analyze, and interpret visual scenes such as humans. The goal of scene understanding is to have a complete understanding of visual scenes by analyzing the context, identifying objects and their relationships, and interpreting the semantic meaning of the scene. This study provides a comprehensive survey of scene understanding, covering a wide range of strategies and methods (Aarthi and Chitrakala, 2017). Many researchers have applied different techniques for scene recognition (Sun et al., 2018). The research presents a complete scene recognition representation by incorporating deep characteristics from three selective views: object meaning, international physical appearance, and context-dependent appearance. The object semantics representation is obtained by a deep learning-based multi-class detection using spatial fisher matrices to store item categorization and pattern characteristics. To collect the contextual information of the scene image, a multi-directional extended temporary memory-based model is used. The initialization of a convolutional neural network’s completely linked layer depicts an overall look of the scene image’. This evaluation has been performed on three different datasets. By adding object semantics to deep learning frameworks increase the computational cost and training time. This demand restricts the scalability of the method due to the resources needed for integrating object semantics with appearance features in deep learning. Wang et al. (2020) presented a unique deep feature fusion method named deep feature fusion through adaptive selective metric learning (DFF-ADML) to study identical reliable data needed for scene recognition. To be more explicit, they create a novel discriminative metric learning problem that not only fully uses discriminative information from each deep feature vector but also adaptively combines complementary information from distinct deep feature vectors. Although the study shows promising outcomes for scene recognition through deep feature fusion and adaptive discriminative metric learning, its effectiveness could be constrained to certain datasets and scenarios. To fully gauge its robustness and applicability, further evaluation across diverse datasets and real-world scenarios is essential (Zeng et al., 2021). This study aims to present a comprehensive assessment of current advancements in scene categorization, including challenges, benchmark datasets, taxonomy, and quantitative performance utilizing deep learning (Yin et al., 2013). This study provided a fuzzy reasoning-based scene semantics identification system. The system has three components: image preprocessing, target recognition, and a fuzzy reasoning machine. In contrast to earlier methods, pattern classifier outputs are fuzzed, fuzzy connections among objectives are obtained, and fuzzy deduction is performed using fuzzy automata. According to the experiment results, this technique might eliminate the problem of patter’s mistaken positive and incorrect negative. This method encounters challenges in determining suitable thresholds for comparisons, which result in false positives and false negatives. Additionally, relying on fuzzy reasoning add complexity to implementation and interpretation, which could impact scalability and generalizability.

Furthermore, López-Cifuentes et al. (2020) offer a unique scene identification, an end-to-end multi-modal CNN technique, that includes image and contextual data via a focused component. Contextual knowledge in the form of semantic segmentation is used to restrict features gathered from a color image using details contained in the semantic depiction: the collection of scene objects and components and their relative placements. By focusing CNN’s responsive fields on them, this restricting technique promotes learning of suggestive scene data and enhances scene recognition. The main drawback highlighted by the author in the study is that while semantic segmentation aids in guiding the scene recognition process with RGB images, any inaccuracies or flaws in the semantic segmentation can negatively impact the overall performance of the proposed method. Seong et al. (2020) used CNN to create a novel scene identification approach. Wang et al., 2024 The proposed technique leverages the CNN framework, FOS Net (fusion of objects and scenario), based on the fusion of object semantics and deep appearance features for scene recognition and scene information in the provided image. Moreover, to train the FOSNet and improve scene identification performance, a unique loss called scene consistency loss (SCL) is being developed. Based on the distinctive qualities of the scene such as how the “sceneness” expands and how the context class remains constant during the picture, the suggested SCL has been developed. This study has limitations in determining the appropriate SCL rate (γ) in the loss function, which can affect the effectiveness of SCL. The impact of SCL on different models and training scenarios needs further investigation to understand and address these challenges effectively (Meena et al., 2022). To begin, the image was represented by a set of local feature areas. Then, based on the new model, the probability discovered among images, local areas, and semantic categories helps to compute the posteriors and recognize the object. The EM algorithm was used to estimate the model’s parameters and failed to capture the irregularly shaped objects or groups of small objects. Conradsen and Nilsson (1987), Wei et al. (2015) and Xu and Wei (2023) proposed a hybrid method for multi-label object recognition that uses a transfer learning-based approach with four separate CNN models and feature fusion, resulting in higher accuracy than existing techniques. The HCP method struggles with scenes containing overlapping or closely positioned objects since it relies on single-shot detection without precise bounding box localization. Additionally, its performance affected by the quality and diversity of training data, potentially limiting its ability to generalize across different multi-label image datasets (Pohlen et al., 2017). The study presents a unique architecture for semantic segmentation in street scenes that is close to ResNet. It combines pixel-level accuracy with multi-scale context, and it achieves an intersection-over-union score of 71.8% on the Cityscapes dataset. The suggested architecture makes use of two processing streams: one that performs pooling operations to provide robust recognition features and the other that carries information at a higher resolution for exact adherence to segment bounds. The limitations underscore challenges related to memory usage, boundary preservation, and computational efficiency in semantic segmentation tasks, which could impact the model’s overall performance and applicability in real-world scenarios (Wang and Yuan, 2016; Zhang et al., 2022). On the PASCAL VOC 2007 and 2012 datasets, the suggested Scene-Object Network for Detection (SOND) model produces competitive results, outperforming the Fast-RCNN baseline by 4.2% on VOC 2007 and 3.4% on VOC 2012, with mean average precision (mAP) scores of 74.2 and 71.8%, respectively. Reducing localization mistakes and improving item identification performance are achieved through the use of enhanced proposals, which are produced by merging suggestions from Edge Box and Selective Search. This methodology enhances the outcomes attained using the SOND model. The proposed method utilizes a combination of Selective Search and Edge Box proposals, which could add complexity and computational overhead to the system.

## 3 Materials and methods

### 3.1 System methodology

**Figure 1:**
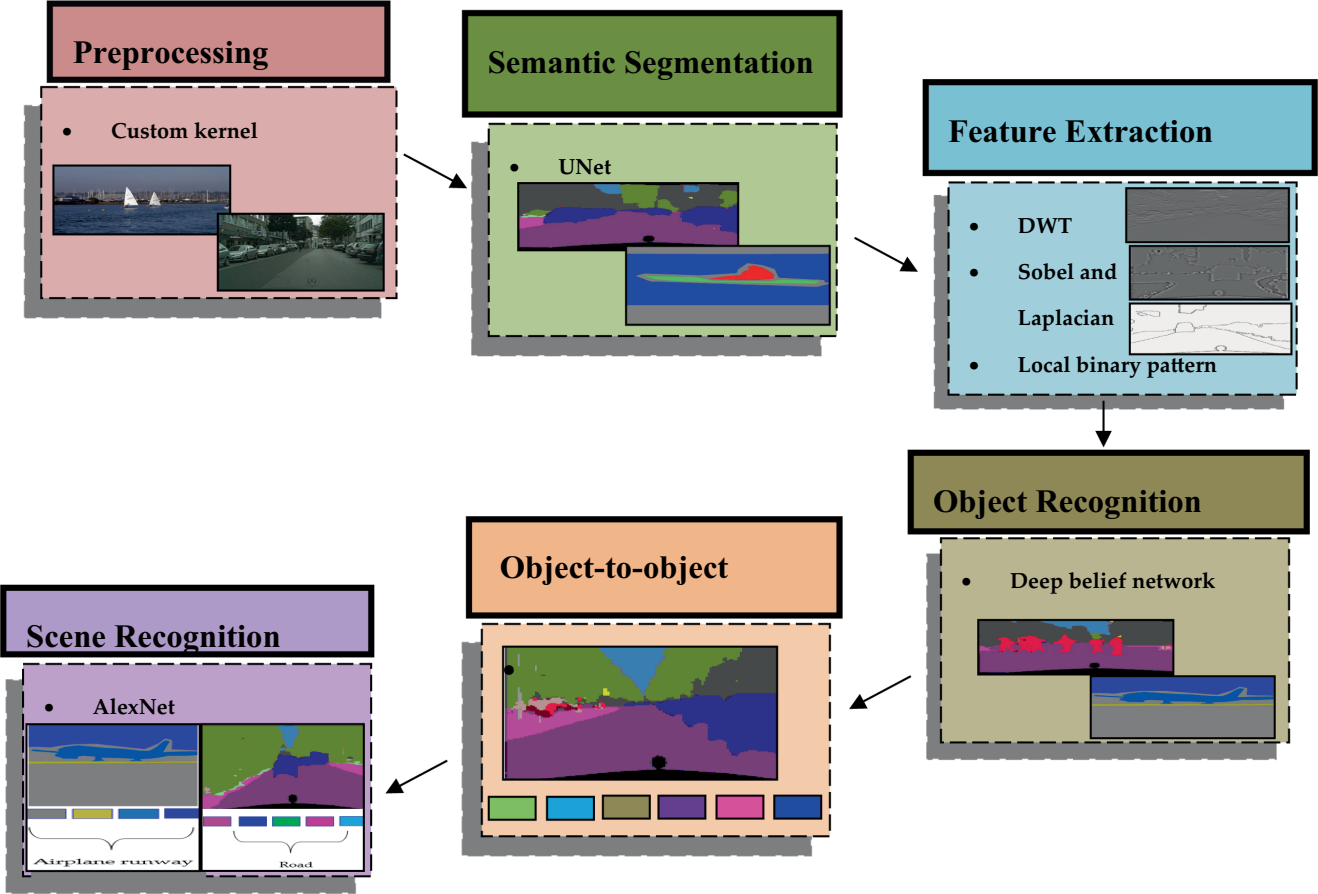
The architecture of the proposed system.

The proposed methodology introduces an innovative approach for multi-object detection and scene recognition utilizing RGB image data. The initial phase involves inputting images into the semantic segmentation process, wherein several objects in the scene are segmented using the UNet model. Subsequently, features of the identified objects (Huang et al., 2022) are extracted using three distinct algorithms: Discrete Wavelet Transform (DWT), Sobel, Laplacian, and textual analysis through Local Binary Pattern (LBP). After that, the deep belief network model uses these properties to recognize various things in the image. The recognized objects undergo analysis for object-to-object relationships. Finally, an AlexNet Neural Network is employed to predict the scene label based on the relationships between the objects. An in-depth discussion about each phase of this procedure is mentioned in the succeeding subsections. Figure 1 depicts the overall architecture of the suggested approach. The proposed system’s architecture is visually represented in Figure 1.

### 3.2 Noise removal

**Figure 2:**
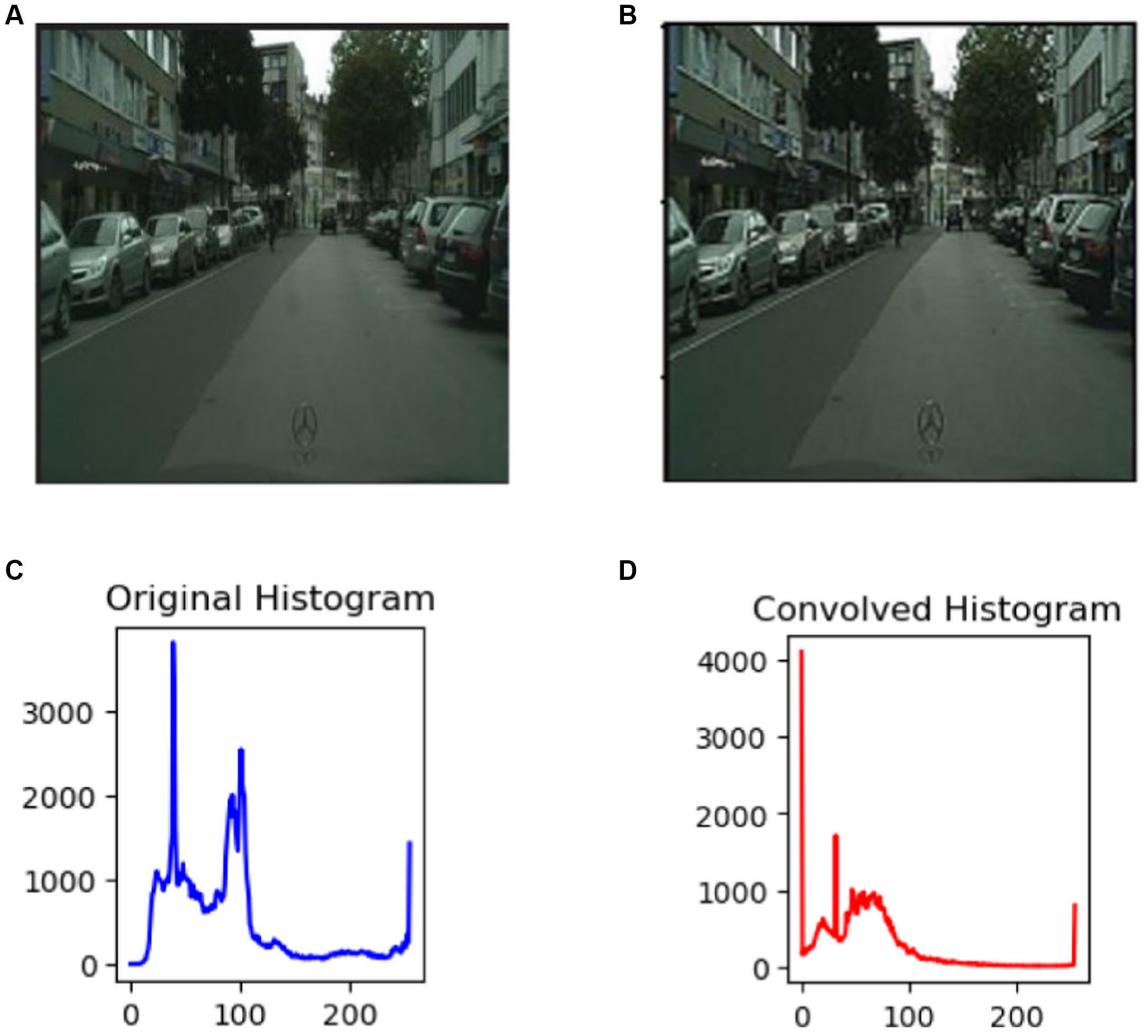
Outcomes of pre-processing images: **(A)** the original picture; **(B)** the filtered image; **(C)** the original image histogram; **(D)** the convolved image histogram.

Noise removal and image smoothing involve eliminating undesired variations or artifacts in an image that do not belong to the underlying scene. It is an important step because it enhances image quality. This process also entails reducing the high-frequency components in the image, resulting in a visually smoother appearance by suppressing abrupt changes and fine details. In the pre-processing phase (Westin et al., 2000; Awate and Whitaker, 2006; Gong et al., 2014; Xu et al., 2022; Chen et al., 2024), the raw images within the datasets are gathered under diverse circumstances, including variations in illumination and contrast distribution, elevated intensity values, and fluctuations in object scales within the images (refer to Figure 2A). To mitigate this undesired information, an initial step involves adjusting the dimensions to 224 × 224 through fixed window resizing. Then, we used custom convolution for sharpening enhanced edge features and details (Qu et al., 2023). Custom convolution kernel is a tiny matrix of numerical values that is used to apply a specific operation to an input image. Convolving the kernel with the image entails sliding the kernel (Xu et al., 2023) across the image and conducting a mathematical operation at each place. This operation computes the weighted sum of the image’s pixel values, with the weights specified by the kernel values (Yin et al., 2020). Figure 2B displays the image following the convolution operation of an enhanced image, whereas Figures 2A,B depict the initial unfiltered image. Additionally, Figures 2C,D display the histograms of the two photos because it is frequently challenging to analyze the differences.

The mathematical formula for a bespoke convolution kernel entails representing the kernel’s weights as a matrix (Sun G. et al., 2019). Let us call the input picture I(a,b) and the convolution kernel K(*m,n*), where *m* and *n* indicate the kernel’s spatial coordinates. At a given pixel (a,b) , the convolution operation is calculated as follows:


$$\left(I*K\right)\left(a,b\right)=\underset{m,n}{\sum }I\left(a-m,b-n\right).K\left(m,n\right)$$

Here, ∗ donates the convolution operation, and summation is performed on the entire kernal region. For 3×3 kernal:


$$K=\left[\begin{array}{ccc} 0 & -1 & 0\\ -1 & 5 & -1\\ 0 & -1 & 0 \end{array}\right]$$

The convolution operation for a pixel (x, y) would be as follows:


$$\begin{array}{l} \left(I*K\right)\;\left(a,b\right)=I\left(a-1,b-1\right).\left(-1\right)+\left(a-1,b\right).\\ \;\;\;\;\;\;\;\;\;\;\;\;\;\;\;\;\;\;\;\;\;\;\;\;\;\;\;\;\;\;\;\;\;\;\;\left(-1\right)+I\left(a-1,b+1\right).0+I\left(a,b-1\right),\\ \;\;\;\;\;\;\;\;\;\;\;\;\;\;\;\;\;\;\;\;\;\;\;\;\;\;\;\;\;\;\;\;\;\;\;\left(-1\right)+I\left(a,b\right).5+I\left(a,b+1\right).\left(-1\right)\\ \;\;\;\;\;\;\;\;\;\;\;\;\;\;\;\;\;\;\;\;\;\;\;\;\;\;\;\;\;\;\;\;\;\;\;+I\left(a+1,b-1\right).0+I\left(a+1,b\right).\left(-1\right)\\ \;\;\;\;\;\;\;\;\;\;\;\;\;\;\;\;\;\;\;\;\;\;\;\;\;\;\;\;\;\;\;\;\;\;\;+I\left(a+1,b+1\right). \end{array}$$

This formula computes the balanced total of pixel values of the surrounding range (x,y) , according to the values of convolution kernal K .

### 3.3 Semantic segmentation

Segmentation is performed to partition an image into meaningful regions or objects, aiding in tasks such as object recognition, image analysis, and compression. It is useful for feature extraction by isolating specific regions of interest, allowing extraction of distinctive features from these segments for further analysis and processing. Following the pre-processing of the photos, each pixel in the image is classified into a distinct class and category using image segmentation (Khan et al., 2024). Segmenting a picture into many parts is known as image segmentation. Semantic segmentation aims to assign a semantic name to each sector after segmenting a picture into meaningful parts (Wang et al., 2022). The main idea is to build a “completely convolutional” network that, by effective inference and learning, can handle indefinitely huge inputs and yield similarly enormous outputs. By converting contemporary classification networks (AlexNet, VGG, and Google Net) into entirely convolutional systems, they can fine-tune the learned representations to the segmentation problem. Chen et al. (2014) demonstrated that these are all connected to the effectiveness of segmentation. Numerous approaches have been looked into to address these issues. Conditional random fields (CRFs) employ greater context in convolutional networks (CNNs) with graphical models to tackle localization problems (Cai et al., 2023; Wang C. et al., 2023; Wang Q. et al., 2023; Zhao et al., 2023). We looked at the “update backgrounds” and the “patch-patch” context (between image sections) (Sun K. et al., 2019). Their model is therefore better at determining the segment borders. A high-quality net (HRNet) tackles the reduction of smooth picture detail in the encoder/decoder-based paradigm (Zhao et al., 2017). This loss happens throughout the encoding procedure. The high-resolution representations are recovered using encoder–decoder models. HRNet, on the other hand, preserves high-resolution representations by occasionally transferring information (Liu H. et al., 2022; Liu Y. et al., 2022; Liu D. et al., 2022; Min et al., 2024; Zhao L. et al., 2024; Zhao X. et al., 2024) between resolutions and connecting the high-to-low convolutions streams in parallel. It is therefore used as the basis for future models and improves segmentation accuracy (Liu et al., 2021; Fu et al., 2023). The multiscale, pyramid network-based Pyramid Scene Parsing Network (PSPN) uses the global context representation of a scene.

**Figure 3:**
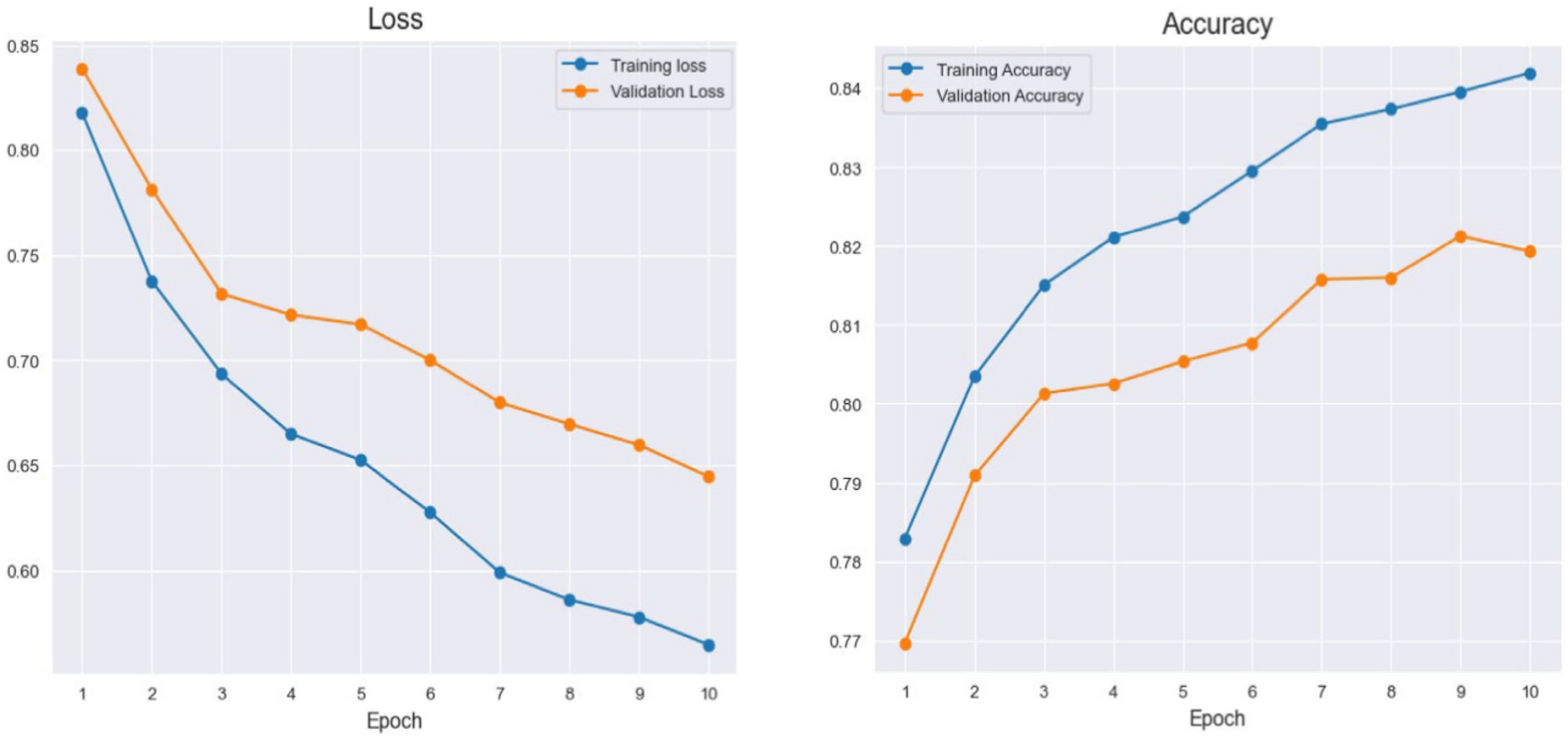
Histogram of Cityscapes performance during training and validation.

The graph depicts the accuracy of training and validation. The y-axis displays the model’s accuracy as a percentage, and the x-axis displays the quantity of training epochs (Jiang et al., 2021; Xiao et al., 2023a,b,c). While the validation loss shows how the data model functions on different data, the training loss measures the variation between the expected and real target values in the training set. It helps the model figure out if it fits the data better or worse. The model’s learning ability from the training data is indicated by the training accuracy, which is displayed in blue. The validation accuracy, indicated in orange, estimates the model’s effectiveness in generalizing new and previously unknown data. Figure 3 illustrates it.

The UNet model is made up of several convolutional blocks for feature extraction and up sampling blocks (Yu et al., 2021; Hou et al., 2023a,b; Xiao et al., 2023a,b,c) for segmentation. Because of the architecture, the model can capture both local and global context information, making it useful for semantic segmentation tasks. The model has been trained to predict pixel-by-pixel semantic labels, which allows it to segment objects and regions in images (Hou et al., 2023a,b). The number of layers and filters in each block (He et al., 2023) enhances the model’s ability to learn hierarchical characteristics and spatial correlations. We have used seven convolution layers (3 in the contracting path, 3 in the expanding path, and 1 in the output layer). The number of filters in these layers’ ranges from 64 to 512, following increasing and decreasing patterns (Hu et al., 2019; Li et al., 2022; Xie et al., 2023) in the contracting and expanding path, respectively. The contracting path (encoding) consists of applying two 3×3 convolutions repeatedly, (with each one being followed by the rectified linear unit activation and a normal kernal initializer), with a 2×2 max pooling operations to downsample the spatial dimensions after each convolution block (Chen et al., 2022). The number of convolutional filters doubles with each downsample, capturing more complex features at multiple scales. After the convolution help, optional dropout layers reduce overfitting by randomly dropping units from the neural network during training. The expansive path (decoding)includes upsampling blocks that consist of a 2×2 transposed convolution that halves the number of feature channels, followed by concatenation with the cropped feature map from the contracting path (Wang C. et al., 2023; Wang Q. et al., 2023; Zhang J. et al., 2024; Zhang X. et al., 2024; Zhao X. et al., 2024; Zhao L. et al., 2024). This convolution is followed by two 3×3 convolutions, each followed by the ReLU activation and normal initialization, which refines the feature map and recover spatial information lost during downsampling (Lyu et al., 2024). The final layer of the model is a 1×1 convolution that maps the feature vector at each pixel to the desired number of classes (Xu et al., 2023). The model uses an Adam optimizer and a sparse categorical cross-entropy loss function, which are suitable for segmentation task (Wu et al., 2023) with non-overlapping labels.

**Figure 4:**
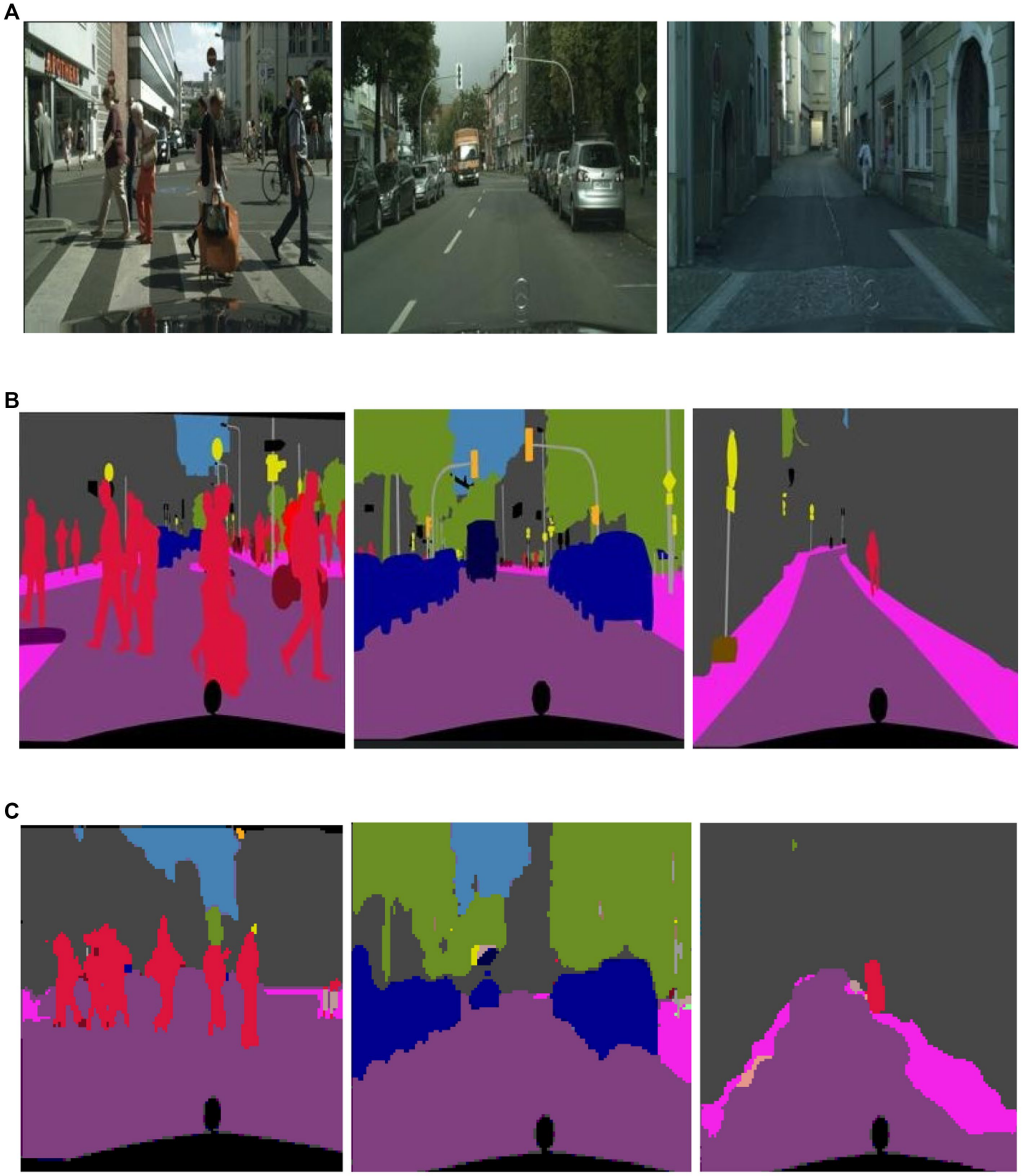
Results of image segmentation on Cityscapes **(A)** original images **(B)** ground truth **(C)** segmented results.

**Figure 5:**
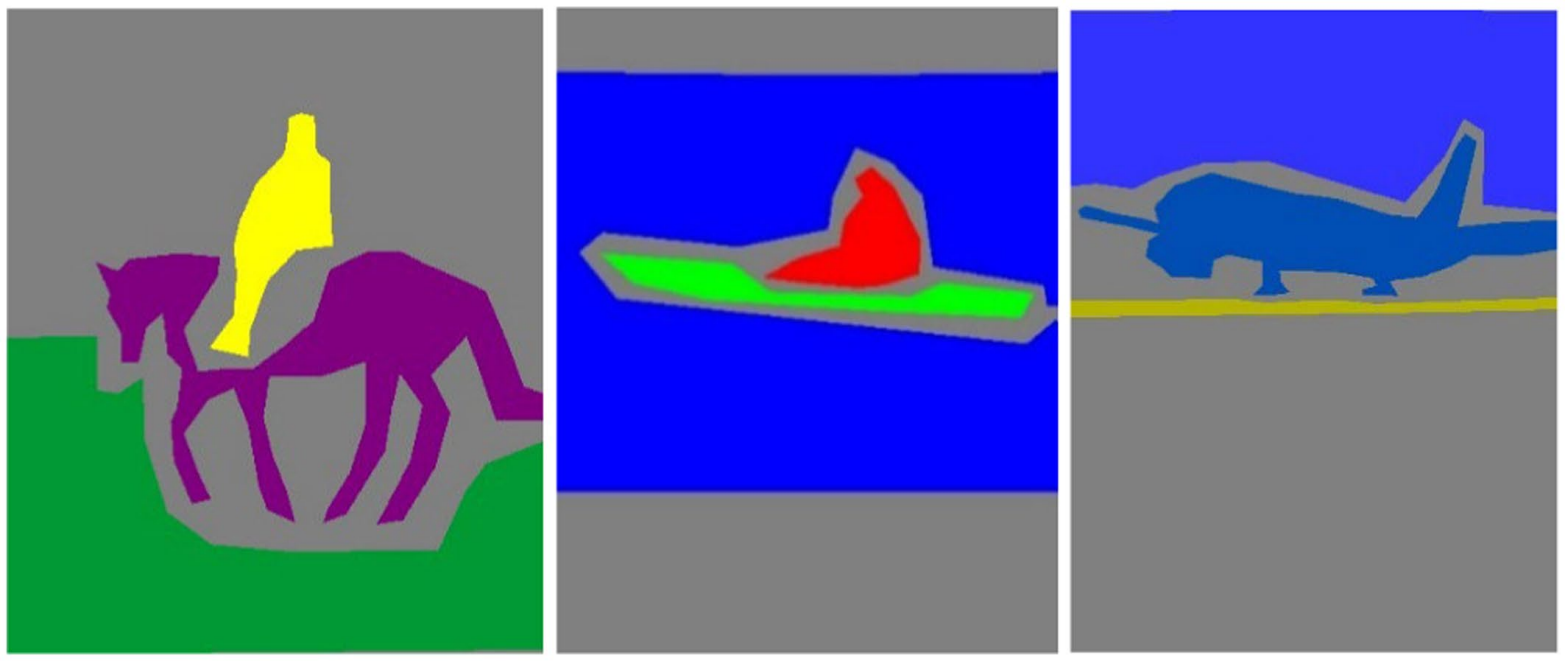
Results of image segmentation on PASCALVOC-12.

During training, the best model weights (Hou et al., 2017; Wu C. et al., 2019; Wu W. et al., 2019; Zhang X. et al., 2024; Zhang J. et al., 2024) are saved using a checkpoint callback based on validation accuracy, which monitors the performance on a validation set separated from the training data (Lu et al., 2022). The network weights are optimized during the training phase to minimize the loss function (Lu et al., 2024) and increase the accuracy of pixel-wise classification (Miao et al., 2023; Mou et al., 2023; Xu et al., 2024). The model is trained using batches of photos with matching segmentation masks, as shown in Figures 4, 5.

### 3.4 Feature extraction

Feature extraction is essential to reduce the complexity of data and highlight important information. By extracting meaningful features, we can enhance the performance of machine learning algorithms and improve accuracy in tasks such as object recognition and classification. Additionally, feature extraction aids in improving the interpretability of models by highlighting meaningful attributes that contribute to decision-making in various applications. Following picture segmentation, extracting object characteristics becomes critical, with each feature playing a specific function in gaining relevant information. The feature extraction procedure employs discrete wavelet transform (DWT) features, Sobel and Laplacian features, and local binary pattern (LBP). In the next subsections, detailed explanations of each feature are provided, along with pseudocode for the whole process, as presented in Algorithm 1.

#### ALGORITHM 1 Object recognition

**Input:**

‘I’: Set of images ‘I = {i1, i2, i3…., in}

**Output:** (n0, n1, …, nN): The classification of each image.

**Initialization:**

D ← []: Image recognition

F ← []: Feature Vector

**Method:**

**For** k = 1 to size (I):

resize_img = imrezise([K], target size): Resize image ‘I[K]’ to a specific ‘target szie’.

seg_img = UNet(resize_img): Apply the UNet segmentation on the images.

**For** s = 1 to size (D):

F ← DWT (D[s]): Apply Discrete Wavelet Transform (DWT) to the segmented region D[s].

F ← Sobel (D[s]): Apply Sobel edge detection to the region D[s].

F ← Laplacian (D[s]): Apply Laplacian filter to the region D[s].

F ← LBP (D[s]): Apply Local Binary Pattern D[s].

Img_class = DBN (F): Recognize the image using Deep Belief Network (DBN) with features ‘F’.

End For

End For

**Return** ‘img_class’ for each image

#### 3.4.1 Discrete wavelet transforms

The discrete wavelet transform (DWT) is discussed as a wavelet-based extension of a finite-energy signal, with a focus on signal representation economy and flawless signal reconstruction (Alessio and Alessio, 2016). An algorithm, for implementing the 2D-DWT feature extraction technique, and the extracted coefficients are utilized to represent the image for classification (Xiao et al., 2023a,b,c; Zheng et al., 2024).

**Figure 6:**
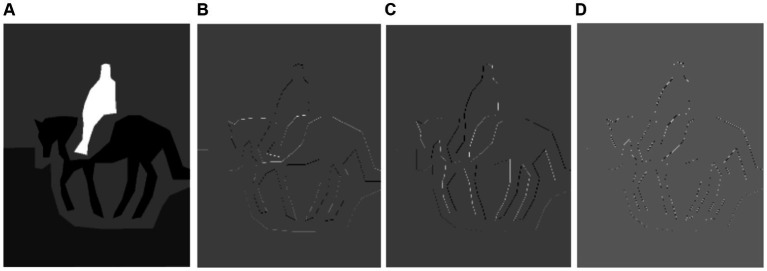
Results of DWT features on PASCALVOC-12 **(A)** grayscale image **(B)** horizontal features **(C)** vertical features **(D)** diagonal features.

**Figure 7:**
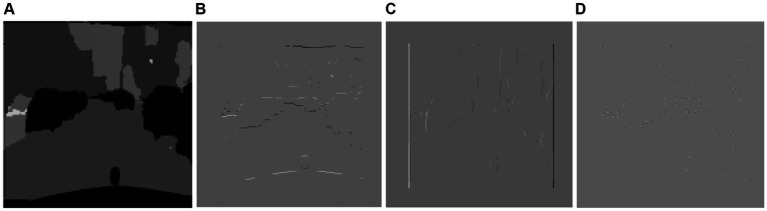
Results of DWT features on PASCALVOC-12. **(A–D)** Show on the Cityscapes.

Discrete wavelet transforms used to analyze images in both the frequency and spatial domains. The DWT recursively decomposes the image into a set of orthogonal wavelet coefficients. This is mathematically accomplished by applying several filters to the image. First, the image is convolved using low-pass and high-pass filters (Hertz et al., 2022; Sheng et al., 2023b; Shi et al., 2023; Fu and Ren, 2024; Qi et al., 2024; Zheng et al., 2024), which represent the division of the image into high-frequency detail and low-frequency approximation components. This process is termed as sub-band coding. The filters used are derived from a selected wavelet function and scaled accordingly. The original image I is thus separated into four sub-images: the approximation coefficients xA , and the detail coefficients xH,xV ,and xD , representing horizontal, vertical, and diagonal details, respectively. The results are presented in Figures 6, 7. Mathematically, it is represented as follows:


$$I\left(a,b\right)=xA\left(\frac{a}{2},\frac{b}{2}\right)+xH\left(\frac{a}{2},\frac{b}{2}\right)+xV\left(\frac{a}{2},\frac{b}{2}\right)+xD\left(\frac{a}{2},\frac{b}{2}\right)$$

where a and b denote pixel coordinates in the two-dimensional image space.

#### 3.4.2 Sobel and Laplacian

The edges in the X and Y axes are recognized in the classic Sobel, and some edge information is lost. To address this, an enhanced Sobel algorithm with an 8-directional template is utilized (Deka and Laskar, 2020). According to the methodology proposed in this research study, Laplacian and Sobel visualization is used to extract information from each pixel in a picture to determine the blurry areas. Next, without the need for picture de-blurring, the blur classes (motion blur or defocus blur) are identified by SVM model training (Yaacoub and Daou, 2019). The design of a fractional order Sobel edge detector is proposed in this study. Sobel gradient operators are used for the first order derivative, while fractional calculus is used for non-integer orders greater than unity.

We employed two distinct edge-detection algorithms to capture the inherent structure and edges within the images. The Sobel operator works by convolving the image with two separate 3×3 kernels which are approximate to the derivatives (Yang B. et al., 2023; Yang D. et al., 2023), one for the horizontal changes and other for the vertical. $G_{X}andG_{y}$ are two images which at each point contain the vertical and horizontal derivative approximations, the combined gradient can be computed as follows:


$$G=G_{x}^{2}+G_{y}^{2}$$

This gradient magnitude corresponds to edge strength of the image at each poi t.

Conversely, the Laplacian of Gaussian is a two-step process that involves smoothing the image. Mathematically, the LoG operator is defined as follows:


$$\nabla^{2}\left[G\left(x,y\right)*I\left(x,y\right)\right]$$

where I(x,y) is the original picture, G(x,y) is the Gaussian filter, and $\forall^{2}$ is the Laplacian function.

**Figure 8:**
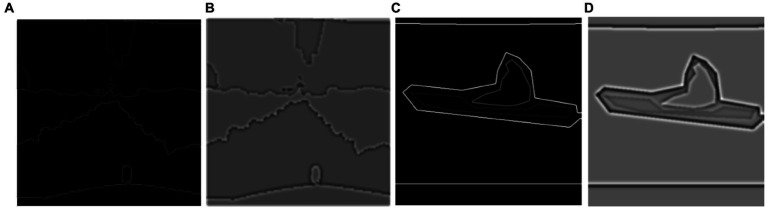
Results of Edge detection over Cityscapes and PASCALVOV-12. **(A)** Shows the Sobel **(B)** shows the Laplacian of Gaussians over Cityscapes **(C,D)** show over PASCALV0C-12.

The Gaussian filter (Sheng et al., 2023a) suppresses noise by smoothing the image, and the subsequent Laplacian filter detects regions of rapid intensity change, thereby highlighting edges. Figure 8 shows the results of the Sobel and Laplacian features.

#### 3.4.3 Local binary pattern

They developed local binary patterns to establish the Hu moment approach. Hu’s 7 moments are computed using the response minima of the proposed local binary pattern (LBP) model, which correspond to the coordination number (CN)* of each contour point of the object. A modified model was incorporated with the local binary patterns corresponding to the coordinate numbers of object contour points to determine the similarity between two binary entities (Kumar and Mali, 2021; Fadaei et al., 2023). Orthogonal Distinction, the local binary pattern, has been improved. The suggested method divides each 33 local window into two groups, extracts local patterns from each group, and provides the feature vector by concatenating group patterns (Pavithra, 2021). A cascaded strategy for content-based image retrieval (CBIR) combining dominant color and uniform local binary pattern (texture) features is proposed in this study. On Wang’s database, with a 75% retrieval accuracy, the method recovers dominating color characteristics at the first level and uniform local binary pattern-based texture information (Shuai et al., 2022) at the second level.

**Figure 9:**
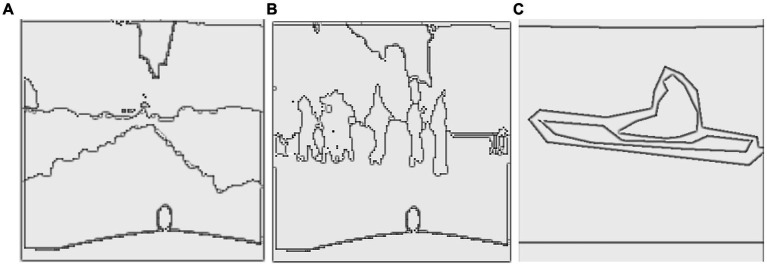
Results of textual pattern **(A)** and **(B)** shows on Cityscapes and **(C)** over the PASCALVOC-12.

By taking into account each pixel and its surrounding neighborhood of radius r, we apply LBP to the gray scale image. We compare the intensity values of each pixel on a circle of radius r with the values of P equally spaced nearby pixels. Neighbors are assigned a binary value of ‘1’ if their intensity is greater than or equal to the center pixel (Yang et al., 2022) and ‘0’ otherwise. The LBP feature picture is created by concatenating these binary digits to create a new binary value, which is then transformed into a decimal integer representing the LBP code for the center pixel. It is calculated as follows:


$$LBP=\left(x_{c},y_{c}\right)=\overset{p-1}{\underset{p=0}{\sum }}2^{p}.1\left(g_{p}-g_{c}\geq 0\right)$$

where LBP value of pixel $x_{c},y_{c}$ . $g_{c}$ is the intensity of the center pixel, $g_{p}$ is the intensity of P equally spaced pixel on the circumference of a circle of radius r around $g_{c}$ , and 1 is the indicator function, equal to 1 if $g_{p}\geq g_{c}$ and 0 otherwise. It is shown in Figure 9.

### 3.5 Multi-object recognition

**Figure 10:**
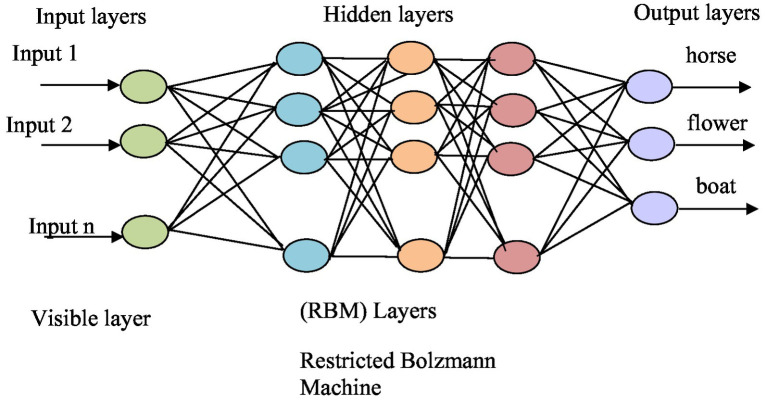
Architecture of deep belief network.

**Figure 11:**
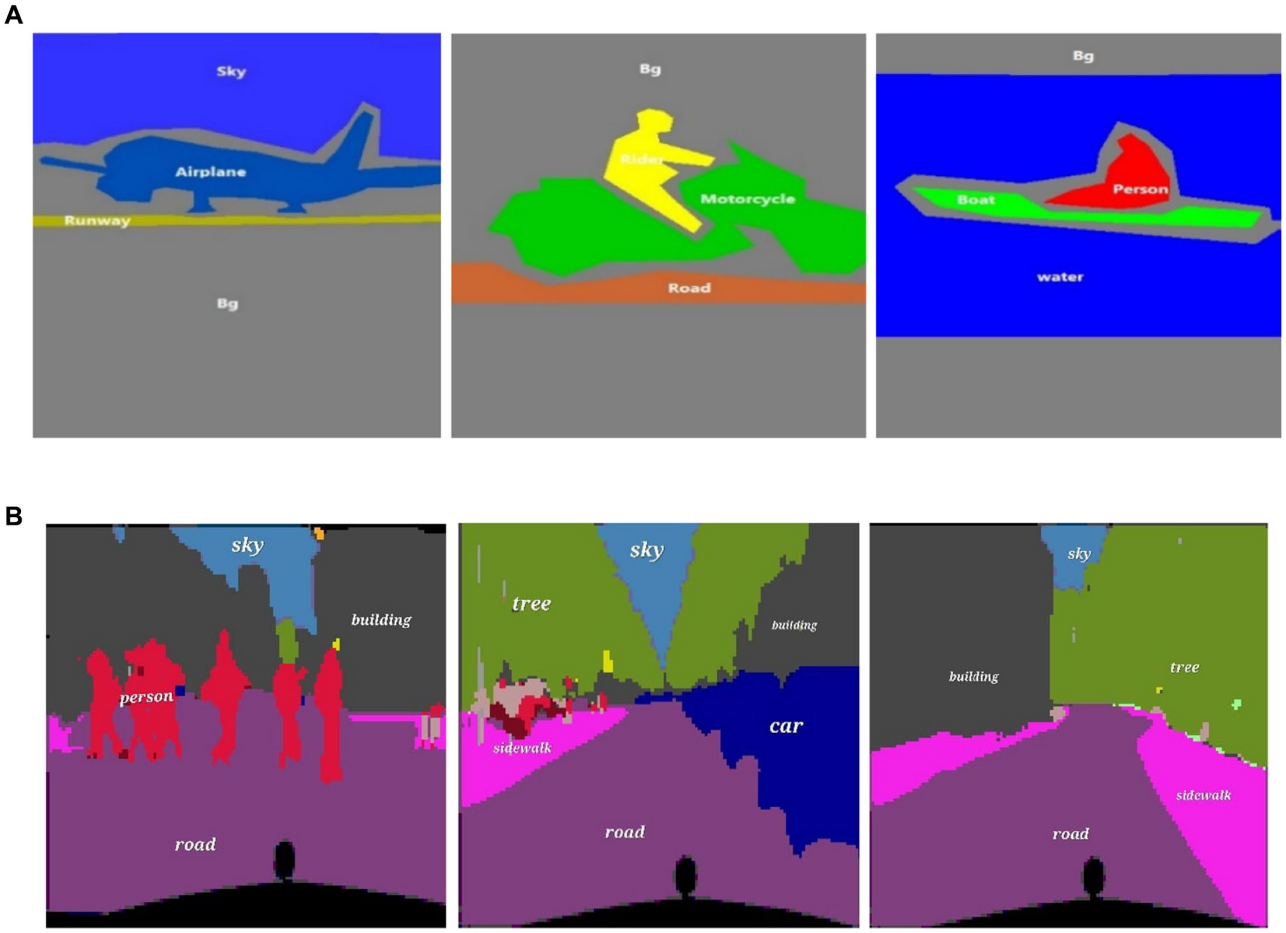
Recognition Results **(A)** shows the PASCALVOC-12 **(B)** over the Cityscape.

Multi-object recognition is crucial because it allows systems to identify and understand multiple objects within an image or scene. This individual object recognition is essential for scene recognition because by recognizing each object separately, we can then understand and recognize the overall scene. We have used deep belief network. This study proposes an object recognition method based on the DBN architecture, primarily for localizing and categorizing objects in photos as Bounding Boxes (B-Box) (Huo et al., 2021). This study offers a neural network model for multi-source heterogeneous iris recognition (MSH-IR) dubbed stacked convolutional deep belief networks-deep belief network (CDBNs-DBN). The model uses a region-by-region extraction technique and positions the convolution kernel through the hidden layer offset to find the effective local texture feature structure. It also employs DBN as a classifier to reduce reconstruction error using the auto-encoder’s negative feedback mechanism. Experimental results on the IIT Delhi iris database recorded by three different iris sensors demonstrate the model’s robustness and identification abilities. Sihag and Dutta (2015) by combining deep belief networks (DBNs) and discrete wavelet transform (DWT), reduces training time and computational complexity in object categorization. The use of DWT facilitates the acquisition of low-resolution pictures, which are subsequently utilized to train multiple DBNs. A weighted voting technique is used to integrate the results of various DBNs. Compared with standard DBNs, the performance of this technique is proven to be competent and faster. Hongmei and Pengzhong (2021) suggested a sparse penalty mechanism for the convolutional restricted Boltzmann machine (CRBM) model that is based on cross-entropy. This mechanism helps to maintain the hidden layer units at a lower activation state and reduces homogeneity of the convolution kernel in the convolutional deep belief network (CDBN). To compensate for the gradient, the proposed model employs a parameter learning technique that blends supervised and unsupervised learning and integrates prior knowledge from the samples. The result is shown in Figure 10 for the multiple object recognition. The results are shown in Figure 11.

The visible layer is v and the hidden layers are $x^{1},x^{2},\ldots .x^{k}$ , where k is the number of hidden layers. The joint distribution over visible and hidden units is given as follows:


$$P\left(v,x^{1},x^{2},\ldots .x^{k}\right)=\frac{1}{Z}\exp\left(-E\left(v,h^{1},h^{2},\ldots .h^{k}\right)\right)$$

where $E\left(v,h^{1},h^{2},\ldots .h^{k}\right)$ is the energy function defined as follows:


$$\begin{array}{l} E\left(v,h^{1},h^{2},\ldots .h^{k}\right)=v^{T}W^{1}h^{1}-h^{1}W^{2}h^{2}-\ldots \\ \;\;\;\;\;\;\;\;\;\;\;\;\;\;\;\;\;\;\;\;\;\;\;\;\;\;\;\;\;\;\;\;\;\;\;\;\;\;\;\;\;\;\;\;\;\;\;\;h^{k-1}W^{k}h^{k}-b^{1}h^{1}-b^{2}h^{2}-\ldots \\ \;\;\;\;\;\;\;\;\;\;\;\;\;\;\;\;\;\;\;\;\;\;\;\;\;\;\;\;\;\;\;\;\;\;\;\;\;\;\;\;\;\;\;\;\;\;\;\;-b^{k}h^{k}-c^{T}v. \end{array}$$

where $W^{i}$ represents the weight matrix between the visible layer and the ith hidden layer. $b^{i}$ and c are the bias terms. Z is the partition function to normalize the distribution.

Given the visible layer and vice versa, the conditional probabilities for the hidden layers are computed as follows:


$$P\left(h^{i}|v\right)=\sigma \left(W^{i}h^{i-1}+b^{i}\right)$$

$$P\left(v|h^{i}\right)=\sigma \left(W^{iT}h^{i}+c\right)$$

where σ is the logistic sigmoid function.

### 3.6 Object-to-object relation

After recognizing the objects within an image, the next step involves identifying the relationships between these objects and how they interact with each other to form a cohesive scene. Understanding these relationships is a key to interpreting the overall context and meaning of the scene. Object-to-object relations are concerned with describing and comprehending the interactions and relationships that exist between particular objects in a given scenario. This analysis frequently considers qualities, spatial arrangements, and functional interdependence to provide insights into how objects connect to one another in a specific scenario, such as an airplane likely to be seen in the sky or run way not in the middle of roads. The OOR between the object of scene sailing their attributes would be boat size (dimension of boat), type (sailboat, motorboat), and color. Peron (sitting or standing, pose), water (texture, color), and spatial arrangement would be the boat maybe located in the water, either floating or anchored. The person could be on the boat, near the shore, or in the water. The spatial arrangements involve the relative positions of the scene’s boat, person, and elements. Functional dependencies include the boat’s reliance on the water for movement and buoyancy. A person may interact with the boat for navigation or recreation. The scene type of bike riding includes (rider, motorbike and road) and airplane runway (airplane, runway, and sky). It is computed as: each object is represented by a vector $v_{i}$ in a feature space. The relation function is defined as $R=v_{i}+v_{j}$ that quantifies the relationship between object i and object j . Then, object relation is computed as follows:


$$M_{ij}=\overset{N}{\underset{i=1}{\sum }}w_{i}.v_{i}$$

where $M_{ij}$ represents the relationship between the object i and another object j . N is the number of features. $w_{i}$ is the weight associated the i−th feature. $v_{i}$ is the feature.

### 3.7 Scene recognition

Scene recognition involves analyzing the relationships between recognized objects to understand the context and layout of a scene. This process allows models to understand how various objects interact within a scene, leading to a deeper comprehension of the overall context and enabling accurate scene recognition. Zhang et al. (2020) introduce an improved HS-AlexNet model for indoor location that combines the advanced AlexNet network model with Harris feature detection to boost generalization and robustness. The model decreases randomness error in complex and changing placement contexts while improving accuracy and speed. It can be integrated with existing visual indoor positioning technologies to improve the positioning system’s accuracy and speed. Hanni et al. (2017) presented a new technique for indoor scene recognition utilizing RGB and depth pictures, reaching an indoor dataset accuracy of up to 94.4%. The suggested deep CNN model beats the GoogLeNet and AlexNet frameworks, achieving a better accuracy of 75.9% on the benchmark NYUv2 dataset. Sun et al. (2016) introduced a novel approach for scene picture classification based on deep image properties obtained from the Alex-Net model and support vector machine. The experimental results show that the deep convolutional neural network (DCNN) can successfully extract image features, enhancing scene image classification and achieving state-of-the-art classification accuracy. Yun-Zhou et al. (2022) addressed the advancement of artificial intelligence technology in China as well as the shortcomings of the template matching model in neural network recognition. It emphasizes the significance of detecting photos that differ from the template and introduces AlexNet, a model that integrates new technological elements and GPU processing acceleration.

**Figure 12:**
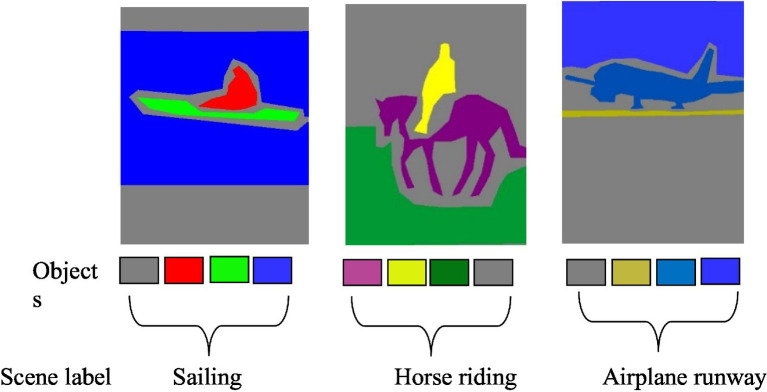
Recognition Results of the PASCALVOC-12 dataset.

**Figure 13:**
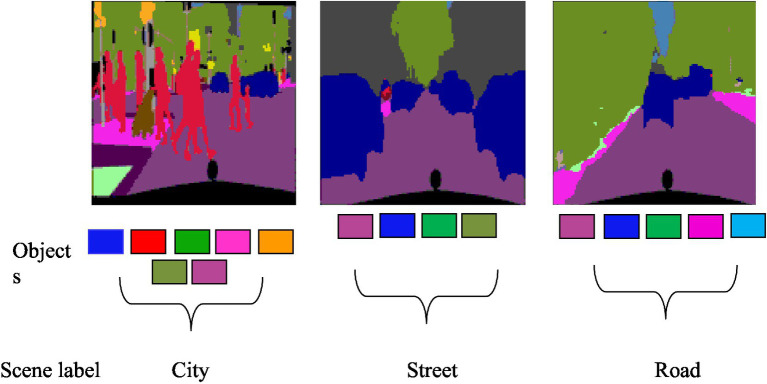
Recognition Results of the Cityscapes dataset.

The final step of the proposed system is the scene recognition. We used AlexNet for the scene recognition. This model consists of specific convolution and fully connected layers. It has an input layer, five convolution layers with ReLU activation, max-pooling layers, and three fully connected layers. The final layer has a number of output nodes which are equal to the specified num classes, and in our case, there were 15 scenes. It takes input images of 224×224 pixels. This model is trained for 20 epochs using the Adam optimizer and cross-entropy loss. The training loop iterates through batches of images, computes the loss, performs back-propagation, and updates the model parameters, as shown in Figures 12, 13. Mathematically, it is computed as follows:

Size of feature map can be calculated as follows:


$$Sizeoffeature\;map=\left[N-f+\frac{2P}{S}\right]+1$$

where N represents the input size, f is the size of convolution kernels, P denotes the padding, and S is the strides.

Convolution layer is expressed as follows:


Conv(X,W,b)=σ(W∗X+b)

where X is the input feature map, W is the convolution filter, b represents the bias term, ∗ denotes the convolution operation, and σ is the ReLU activation function.

ReLU activation function:


ReLU(X)=max(0,X)

Max pooling operation:


$$MaxPool\left(X\right)=\left(X,pool_{size},stride\right)$$

Fully connected layer operations:


FC(X,W,b)=σ(W.X+b)

X is the flattened input vector, W is the weight matrix, and b is the bias term.

Cross Entropy class:


$$CE\left(Y,\hat{Y}\right)=-\sum i\left(Y_{i}.\log(\;{\hat{Y}}_{i})\right)$$

Adam optimizer:


$$m\leftarrow \beta_{1}.m+\left(1-\beta_{1}\right).\nabla_{\theta }J\left(\theta \right)$$

$$v\leftarrow \beta_{2}.v+\left(1-\beta_{2}\right).{\left(\nabla_{\theta }J\left(\theta \right)\right)}^{2}$$

$$\theta \leftarrow \theta -\frac{\alpha }{\sqrt{v}+\varepsilon }.m$$

where m is the first-order moment estimate, v is the second-order moment estimate, $\beta_{1}$ and $\beta_{2}$ are decay rates, α is the learning rate, andε is a small constant to prevent division by zero.

## 4 Experimental setup and datasets

### 4.1 Experimental setup

The three publicly accessible datasets that were utilized to validate the suggested system are described in this section. The overview is followed by the specifics of the implementation and the outcomes of several tests conducted in the three datasets. Caltech 101, Cityscapes, has been utilized for object identification, and PASCALVOC-12 has been utilized for scene recognition. The cross-validation technique has been used to assess the suggested system. Every subject serves as the test set only once in this strategy. It is a unique type of k-fold cross-validation where the number of folds is the same as the total number of dataset instances. Python was used for all processing and experimentation on Windows 11 operating system with 16 GB of RAM, a core i7 processor, and an Intel(R) UHD GPU.

### 4.2 Dataset description

In the subsequent subsection, we provide comprehensive and detailed descriptions of each dataset used in our study. Each dataset is thoroughly introduced, highlighting its unique characteristics, data sources, and collection methods.

#### 4.2.1 The Cityscapes dataset

The Cityscapes dataset (Cordts et al., 2016) represents an extensive database devoted to the semantic interpretation of urban street scenes. The dataset consists of instance-wise, dense, and semantic annotations for pixels in 30 different classes, which are then further categorized into 8 broad categories: objects, flat surfaces, people, cars, buildings, nature, sky, and nothingness. The technique of gathering data comprised taking pictures in 50 cities for several months in a variety of daytime situations with ideal weather. The frames, which were originally captured in video format, were carefully chosen to highlight particular elements including a large variety of dynamic objects, various scenario layouts, and varied backgrounds.

#### 4.2.2 The PASCALVOC-12 dataset

The PASCALVOC-12 dataset (Shetty, 2016) encompasses 21 distinct classes, specifically emphasizing urban street scenes for semantic scene understanding. The dataset comprises 17,000 images capturing diverse and complex scenarios, including both indoor and outdoor settings. The object classes in the dataset include, but are not limited to, person, car, potted plant, motorbike, bicycle, bird, airplane, boat, bottle, bus, cat, dog, chair, cow, dining table, horse, sheep, sofa, and TV/monitor. Moreover, this dataset poses the challenge of illumination variation and motion blur.

#### 4.2.3 The Caltech-101 dataset

Images from Caltech-101 (Kinnunen et al., 2010) classes and one class were dedicated to background clutter make up Caltech-101. There is just one labeled object per image. There are between 40 to 800 photos in each class, for approximately 9,000 photos. Images are available in a wide range of sizes, with 200 to 300 pixels serving as the typical edge length. Cougar, brontosaurus, sidewalk, chair, motorbike, aeroplane, dalmatian, dolphin, faces, ketch, water and tree are among the objects taught in Caltech-101.

## 5 Results and analysis

In this section, we performed different experiments for the proposed system. The system is evaluated using different matrices, including confusion matrix, precision, recall, F1 score, and receiver operating characteristic (ROC) curve. The detailed discussion and analysis are described below.

### 5.1 Object recognition accuracies

Across the tests, our suggested method outperformed the others in terms of accuracy across all three datasets. Confusion matrices are used to illustrate the recognition accuracy obtained for each of the three dataset classes. A classifier’s performance is summed up in a confusion matrix by true, false, and negative positives and negatives. The diagonal of the matrix displays the quantity of true positives or correctly identified classes. The confusion matrices for the Cityscapes, PASCALVOC-12, and Caltech 101datasets are shown in Tables 1–3.

**Table 1:** Object recognition accuracy over the Cityscapes dataset.

Obj	B1	BG	BS	CR	MK	PS	RD	SK	SW	TR	TN
BI	0.96	0.00	0.00	0.00	0.00	0.00	0.02	0.00	0.00	0.02	0.00
BG	0.00	0.96	0.00	0.01	0.00	0.00	0.00	0.03	0.00	0.00	0.01
BS	0.00	0.00	0.94	0.00	0.00	0.00	0.00	0.05	0.00	0.01	0.00
CR	0.00	0.00	0.00	0.98	0.00	0.00	0.02	0.00	0.00	0.00	0.00
MK	0.01	0.00	0.01	0.00	0.91	0.00	0.00	0.05	0.00	0.02	0.00
PS	0.00	0.00	0.00	0.00	0.00	0.97	0.00	0.00	0.00	0.03	0.00
RD	0.00	0.00	0.03	0.00	0.00	0.00	0.96	0.00	0.01	0.00	0.00
SK	0.00	0.00	0.01	0.01	0.00	0.00	0.00	0.97	0.00	0.01	0.00
SW	0.00	0.02	0.00	0.00	0.01	0.00	0.00	0.00	0.97	0.00	0.00
TR	0.00	0.00	0.00	0.00	0.01	0.00	0.02	0.00	0.00	0.97	0.00
TN	0.00	0.01	0.00	0.00	0.03	0.00	0.00	0.00	0.00	0.00	0.96
Recognition accuracy = 95.90%											

Confusion matrix Cityscape dataset, BI, bicycle, BG, building, BS, bus, CR, car, MB, motorbike, PS, person, RD, road, SK, sky, SW, sidewalk, TR, tree, TN, train.

**Table 2:** Object recognition accuracy over the Caltech 101 dataset.

Obj	AE	BR	CS	CH	CG	DM	DP	FS	KT	MB
AE	0.99	0.00	0.00	0.00	0.00	0.00	0.01	0.00	0.00	0.00
BR	0.00	0.85	0.08	0.00	0.04	0.02	0.00	0.00	0.00	0.01
CS	0.00	0.00	0.98	0.00	0.00	0.01	0.00	0.01	0.00	0.00
CH	0.00	0.06	0.00	0.86	0.01	0.01	0.00	0.00	0.00	0.06
CG	0.00	0.03	0.00	0.00	0.90	0.00	0.05	0.00	0.02	0.00
DM	0.05	0.00	0.00	0.00	0.02	0.84	0.05	0.00	0.03	0.01
DP	0.00	0.00	0.01	0.00	0.00	0.00	0.92	0.00	0.03	0.04
FS	0.00	0.00	0.03	0.00	0.00	0.00	0.00	0.97	0.00	0.00
KT	0.03	0.00	0.00	0.02	0.00	0.00	0.01	0.02	0.92	0.00
MB	0.00	0.00	0.00	0.00	0.01	0.00	0.00	0.00	0.00	0.99
Recognition accuracy = 92.2%										

Confusion matrix Caltech 101 dataset; AE, aeroplane; BR, brontosaurus; CS, casrside; CH, chair; CG, cougar; DM, dalmatian; DP, dolphin; FS, Faces; KT, ketch; MO, motorbike.

**Table 3:** Object recognition accuracy over the PASCALVOC-12 dataset.

Obj	AP	BC	BD	BL	BS	BT	CH	CR	CT	CW	DG	DT	HE	MB	TN	PP	PR	SF	SH	TV
AP	1	0.00	0.00	0.00	0.00	0.00	0.00	0.00	0.00	0.00	0.00	0.00	0.00	0.00	0.00	0.00	0.00	0.00	0.00	0.00
BC	0.0	0.97	0.00	0.00	0.00	0.00	0.00	0.00	0.00	0.00	0.00	0.00	0.03	0.00	0.00	0.00	0.00	0.00	0.00	0.00
BD	0.0	0.00	0.96	0.00	0.00	0.00	0.00	0.00	0.00	0.03	0.00	0.00	0.00	0.00	0.00	0.00	0.00	0.00	0.00	0.00
BL	0.0	0.02	0.00	0.77	0.00	0.00	0.00	0.00	0.03	0.00	0.07	0.00	0.05	0.00	0.00	0.00	0.00	0.05	0.00	0.00
BS	0.0	0.00	0.00	0.00	0.98	0.00	0.00	0.00	0.00	0.00	0.00	0.02	0.00	0.00	0.00	0.00	0.00	0.00	0.00	0.00
BT	0.0	0.00	0.00	0.00	0.00	0.97	0.00	0.00	0.00	0.00	0.02	0.00	0.00	0.00	0.00	0.01	0.00	0.00	0.00	0.00
CH	0.0	0.00	0.02	0.00	0.00	0.00	0.97	0.00	0.00	0.00	0.00	0.00	0.00	0.01	0.00	0.00	0.00	0.00	0.00	0.00
CR	0.0	0.00	0.00	0.03	0.00	0.00	0.00	0.87	0.00	0.00	0.04	0.00	0.00	0.00	0.03	0.00	0.00	0.00	0.03	0.00
CT	0.0	0.00	0.00	0.00	0.00	0.00	0.00	0.00	1	0.00	0.00	0.00	0.00	0.00	0.00	0.00	0.00	0.00	0.00	0.00
CW	0.0	0.00	0.00	0.00	0.00	0.00	0.00	0.00	0.00	1	0.00	0.00	0.00	0.00	0.00	0.00	0.00	0.00	0.00	0.00
DG	0.0	0.04	0.00	0.00	0.01	0.00	0.00	0.00	0.00	0.00	0.96	0.00	0.00	0.00	0.00	0.00	0.00	0.00	0.00	0.00
DT	0.0	0.01	0.00	0.00	0.00	0.00	0.00	0.00	0.02	0.00	0.00	0.97	0.00	0.00	0.00	0.00	0.00	0.00	0.00	0.00
HS	0.0	0.00	0.00	0.00	0.00	0.02	0.00	0.00	0.00	0.00	0.00	0.00	0.98	0.00	0.00	0.00	0.00	0.00	0.00	0.00
MB	0.0	0.00	0.00	0.00	0.00	0.00	0.00	0.03	0.00	0.00	0.00	0.00	0.00	0.97	0.00	0.00	0.00	0.00	0.00	0.00
TN	0.5	0.00	0.05	0.00	0.02	0.05	0.00	0.00	0.03	0.05	0.00	0.05	0.00	0.05	0.60	0.00	0.00	0.03	0.00	0.02
PP	0.0	0.00	0.00	0.00	0.00	0.00	0.00	0.00	0.00	0.02	0.00	0.00	0.00	0.00	0.00	0.98	0.00	0.0	0.00	0.00
PR	0.0	0.00	0.00	0.00	0.00	0.03	0.00	0.00	0.00	0.00	0.00	0.00	0.00	0.00	0.00	0.00	0.97	0.00	0.00	0.00
SF	0.0	0.02	0.00	0.00	0.00	0.00	0.00	0.00	0.00	0.01	0.00	0.00	0.00	0.00	0.00	0.00	0.00	0.97	0.00	0.00
SH	0.0	0.00	0.00	0.00	0.00	0.00	0.00	0.00	0.00	0.00	0.00	0.00	0.00	0.00	0.00	0.00	0.00	0.00	1	0.00
TV	0.0	0.00	0.00	0.00	0.00	0.00	0.02	0.00	0.00	0.00	0.00	0.00	0.00	0.01	0.00	0.00	0.00	0.02	0.00	0.97
Recognition accuracy = 96%																				

Confusion matrix PASCALVOC-12 dataset; AP, aeroplane; BC, bicycle; BD, bird; BE, bottle; BI, bicycle; BS, bus; BT, boat; CH, chair; CR, car; CT, cat; CW, cow; DG, dog; DT, dinnintable; HS, horse; MB, motorbike; TN, train; PP, pottedplant; PR, person; SF, sofa; SH, sheep; TV, television/monitor. Receiver Operating Characteristic Curves for Cityscapes; PASCALVOC-12 and Caltec-101 dataset.

### 5.2 Receiver operating characteristic curve for Cityscapes, PASCALVOC-12, and Caltech-101 dataset

**Figure 14:**
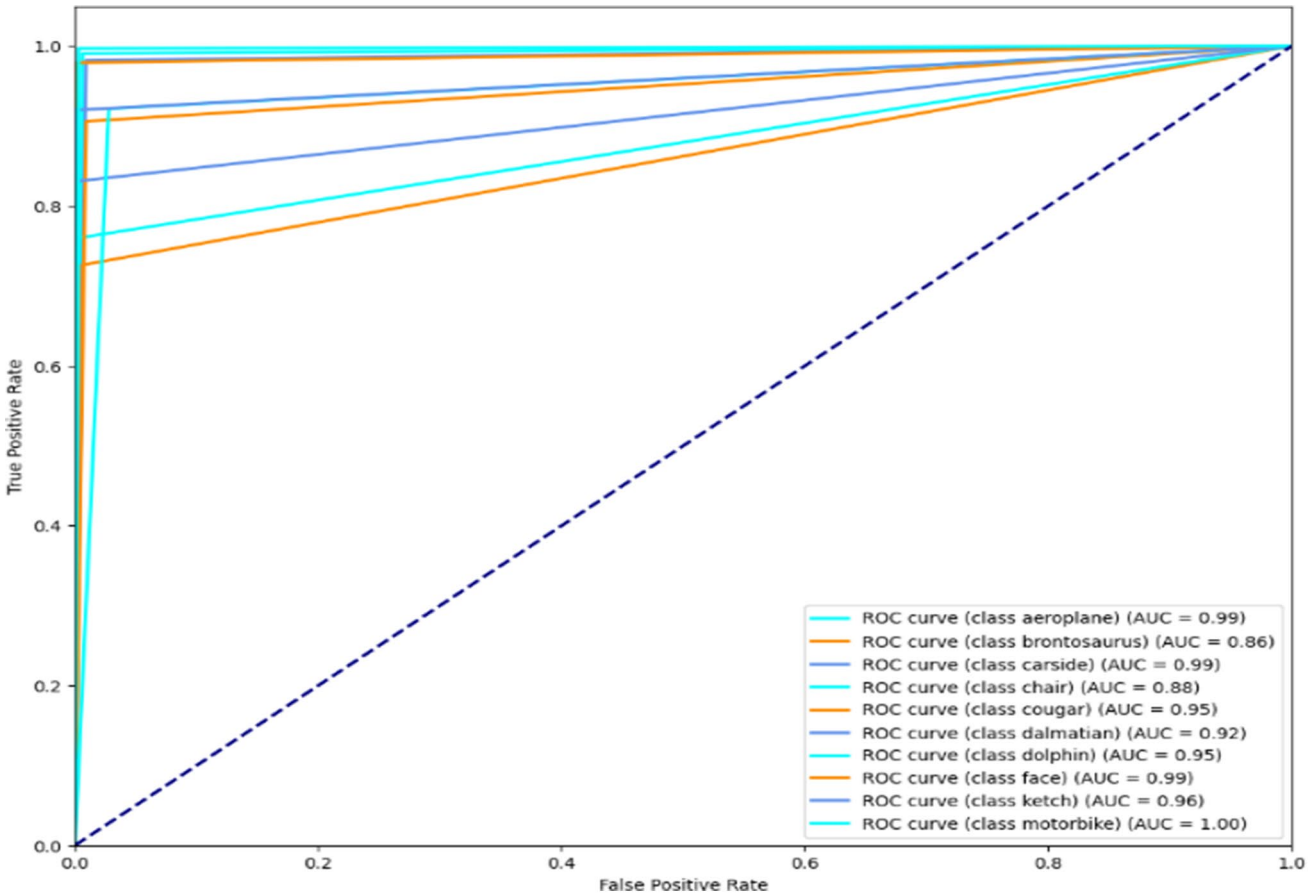
ROC curve over the Caltech dataset.

**Figure 15:**
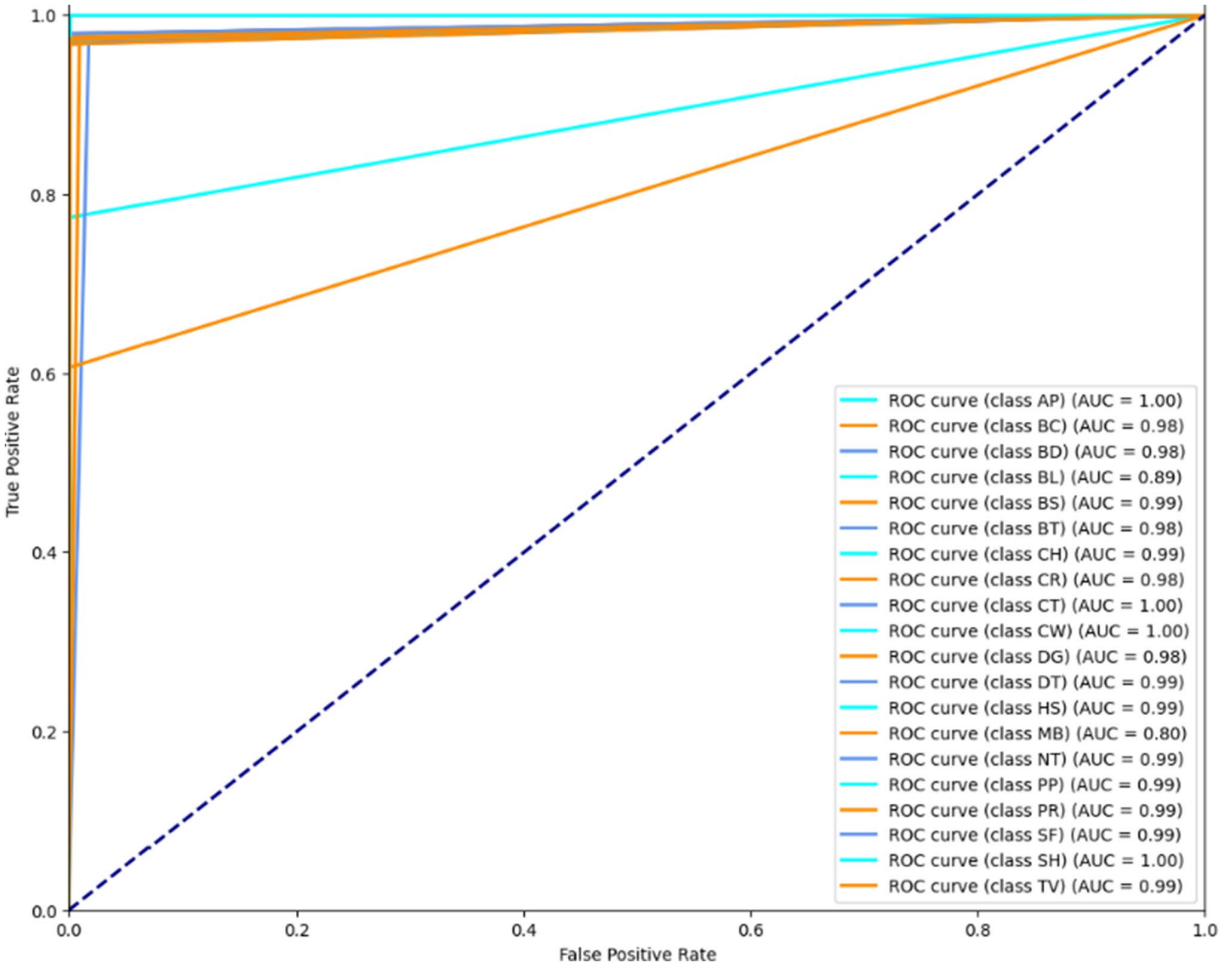
ROC curve over the PASCALVOC-12 dataset.

**Figure 16:**
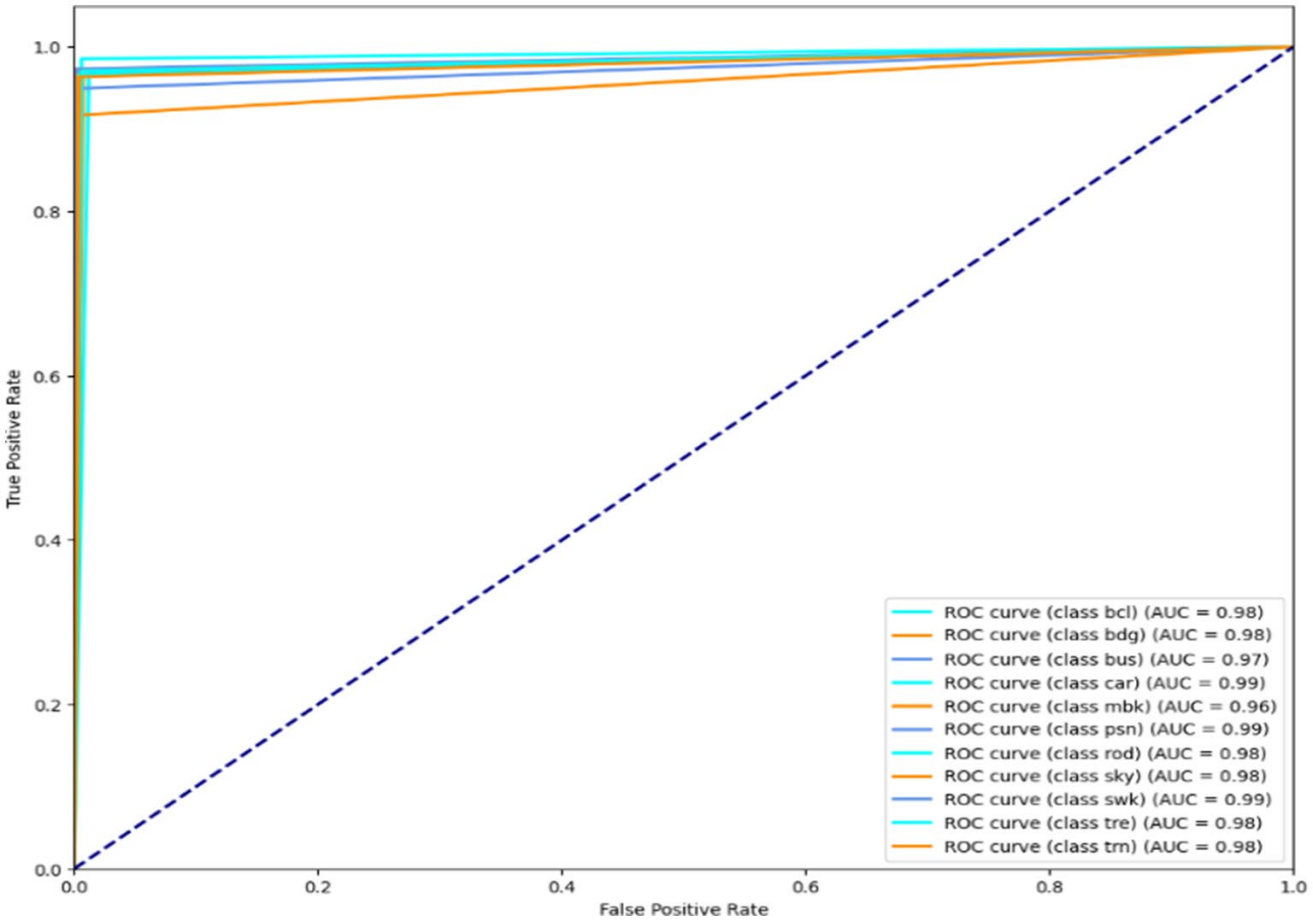
ROC curve over the Cityscape dataset.

Illustration of a binary classification model’s performance at various classification thresholds is called a Receiver Operating Characteristic (ROC) curve. It plots the genuine positive rate (sensitivity) against the false positive rate (1-specificity) for different threshold settings. It can evaluate and contrast model performance since it visually depicts a model’s capacity to distinguish between positive and negative scenarios across various threshold values. The results are shown in Figures 14–16. It is calculated as follows:


$$Sensitivity\left(TruePositiveRate\right)=\frac{TruePositives}{TruePositive+FalseNegatives}$$

$$FalsePositiveRate=\frac{FalsePositives}{FalsePositive+TrueNegatives}$$

### 5.3 Scene recognition accuracy

In this experimental setup, we extracted the scenes recognition. The experimentation involved utilizing the PASCALVOC-12 datasets. We extracted the 15 scenes over the PASCALVOC-12, and Table 4 shows the confusion matrix for scene recognition on the PASCALVOC 112 dataset. The experiment with 30 iterations resulted in an average recognition accuracy of 97.32%.

**Table 4:** Scene recognition achieved over the PASCALVOC-12 dataset.

Scn	AI	BE	BI	CE	CY	FL	FO	HO	PA	PL	RO	SA	SE	SP	TR
AI	96.30	3.70	0.00	0.00	0.00	0.00	0.00	0.00	0.00	0.00	0.00	0.00	0.00	0.00	0.00
BE	2.50	97.50	0.00	0.00	0.00	0.00	0.00	0.00	0.00	0.00	0.00	0.00	0.00	0.00	0.00
BI	0.00	0.00	96.49	0.00	0.00	0.00	0.00	0.00	0.00	0.00	0.00	0.00	0.00	0.00	0.00
CI	0.00	0.00	0.00	84.85	15.15	0.00	0.00	0.00	0.00	0.00	0.00	0.00	0.00	0.00	0.00
CY	0.00	0.00	0.00	0.00	98.28	0.00	0.00	0.00	0.00	0.00	0.00	0.00	0.00	0.00	0.00
FL	0.00	0.00	0.00	0.00	0.00	96.97	0.00	0.00	0.00	0.00	0.00	1.72	0.00	0.00	0.00
FO	0.00	0.00	0.00	0.00	0.00	0.00	1	0.00	0.00	0.00	0.00	0.00	0.00	0.00	0.00
HO	0.00	0.00	0.00	0.00	0.00	0.00	0.00	98.33	0.00	0.00	0.00	0.00	0.00	1.67	0.00
PA	0.00	0.00	0.00	0.00	0.00	0.00	0.00	0.00	98.46	0.00	0.00	0.00	0.00	1.54	0.00
PL	0.00	0.00	0.00	0.00	0.00	0.00	0.00	1.12	0.00	97.75	0.00	0.00	0.00	1.12	0.00
RO	0.00	0.00	0.00	0.00	0.00	0.00	0.00	0.00	0.00	0.00	1	0.00	0.00	0.00	0.00
SA	0.00	0.00	0.00	0.00	0.00	0.00	0.00	0.00	0.00	0.00	0.00	96.49	1.75	0.00	0.00
SE	0.00	0.00	0.00	0.00	0.00	0.00	0.00	0.00	0.00	0.00	0.00	0.00	1	0.00	0.00
SP	0.00	0.00	0.00	0.00	0.00	0.00	0.00	0.00	0.00	0.00	0.00	0.00	0.00	1	0.00
TR	0.00	0.00	0.00	0.00	0.00	0.00	0.00	0.00	0.00	0.00	1.59	0.00	0.00	0.00	98.41
Scene recognition accuracy = 97.32%															

Confusion matrix PASCALVOC-12 dataset; AI, airplane runway; BE, beach; BI, bike riding; CI, city; CY, cycling; FL, flying plane; FO, forest; HO, horse riding; PA, parking; PL, plants; RO, road traffic; SA, sailing; SE, sea; SP, sport; TR, train.

### 5.4 The dataset’s results in terms of F1-score, specificity, and precision

In this experimental study, we assessed the effectiveness of the proposed system by conducting a comparative analysis with the AlexNet model. The evaluation of performance was carried out based on precision, recall, and F1-score metrics. The comparative results are presented in Table 5, showcasing the performance of scene recognition over the PASCALVOC-12 dataset. The outcomes obtained over the PASCALVOC-12 dataset are shown in Table 6.

**Table 5:** A comparison of proposed system with other SOTA methods.

Methods	Accuracy %		
	PASCALVOC-12	Cityscape	Caltech 101
Jalal et al. (2021)	93.53		89.26
Rafique et al. (2023)	87.57		88.60
Guo and Gould (2015)	70.7	–	
Hussain et al. (2020)	–	90.13	–
Khodabandeh et al. (2019)	81.80	–	–
Wei et al. (2015)	85.6	–	
Wang et al. (2018)	–	80.1	–
Wu C. et al. (2019) and Xie et al. (2019)	–	–	87.24
Thitisiriwech et al. (2022)	–	78.86	–
Proposed	96	95.90	92.2

**Table 6:** Measurement of the PASCALVOC-12 dataset in terms of precision, specificity, and F1-score.

Scenes	Precision	Sensitivity	Specificity	F1-Score	Scenes	Precision	Sensitivity	Specificity	F1-Score
AI	0.98	0.96	1.00	0.97	PA	1.00	0.98	1.00	0.98
BE	0.91	0.97	1.00	0.94	PL	0.99	0.98	1.00	0.98
BI	1.00	0.96	1.00	0.98	RO	0.95	1.00	1.00	0.98
CI	1.00	0.85	1.00	0.92	SA	1.00	0.96	1.00	0.98
CY	0.92	0.98	0.99	0.95	SE	0.98	1.00	1.00	0.99
FL	1.00	0.97	1.00	0.98	SP	0.93	1.00	1.00	0.96
FO	0.95	1.00	1.00	0.97	TR	1.00	0.98	1.00	0.99
HO	0.98	0.98	1.00	0.98	Mean	0.98	0.98	1.00	0.98

AI, airplane runway; BE, beach; BI, bike riding; CI, city; CY, cycling; FL, flying plane; FO, forest; HO, horse riding; PA, parking; PL, plants; RO, road traffic; SA, sailing; SE, sea; SP, sport; TR, train.

### 5.5 Comparison with other state-of-the-art methods

The recognition accuracies attained by the suggested system have been compared with contemporary approaches that have been assessed on the same three testing datasets. Table 5 presents the outcomes of the suggested system compared with other SOTA object identification and scene comprehension techniques assessed on one or more of the three datasets utilized in this study. The accuracy scores demonstrate how much better the suggested system performs than any of them.

## 6 Discussion

The scene recognition framework starts by improving the quality of input images using special techniques. It then identifies different objects within the images through a process of UNet segmentation technique. Next, it extracts important features such as edges and textures from these objects using methods such as Discrete Wavelet Transform. These features help achieve high accuracy in recognizing scenes. A deep belief neural network is used to recognize multiple objects, followed by analyzing how these objects relate to each other. Finally, an AlexNet model assigns labels to scenes based on the recognized objects.

This system utilizes three distinct datasets: PASCALVOC-12, Cityscapes, and Caltech- 101. For the scene recognition task on the PASCALVOC-12 dataset, our analysis encompasses 15 diverse scenes, namely, airplane runway, beach, bike riding, city, cycling, flying plane, forest, horse riding, parking, plants, road traffic, sailing, sea, sport, and train. Within the Cityscapes dataset, our recognition efforts extend to 11 classes, namely, bicycle, building, bus, car, motorbike, person, road, sky, sidewalk, tree, and train. Finally, the recognition task on the Caltech 101 dataset focuses on 10 distinct classes, namely, aeroplane, brontosaurus, carside, chair, cougar, dalmatian, dolphin, faces, ketch, and motorbike. This method works well across different datasets, achieving high recognition accuracies of 96, 95.90, and 92.2% on PASCALVOC-12, Cityscapes, and Caltech-101 datasets, respectively. These results show that the proposed method is effective compared with other SOTA techniques, as shown in Table 5.

## 7 Conclusion

In this study, we developed a segmentation method based on UNet to identify multiple objects in images. Our model accurately recognizes objects in complex environments using three benchmark datasets, namely, PASCALVOC-2012, Cityscapes, and Caltech 101. We start by preprocessing input images, followed by segmenting them and extracting features using techniques such as Discrete Wavelet Transform, Sobel, Laplacian of Gaussian, and Local Binary Pattern. The objects are classified into different classes using a deep belief network (DBN). Finally, we find the relationships between objects and predict scene labels using AlexNet. Our approach performs exceptionally well in terms of accuracy, F1-score, specificity, sensitivity, and ROC curves. Despite its success, we encountered several limitations while working with this model.

## 8 Research limitation

The scene recognition framework has some limitations that need to be addressed for future improvements. A significant challenge involves handling the complex and cluttered backgrounds present in datasets such as Pascal VOC 2012 and Cityscapes, which are more intricate compared with Caltech-101. The model struggles with objects that are hidden or look similar to each other because these datasets contain messy and complex information. Additionally, combining classical feature extraction methods with deep learning techniques was difficult in achieving exceptional object recognition and scene recognition accuracy, especially with diverse and intricate scenes.

In the future, we aim to improve object and scene recognition by implementing different deep learning techniques to overcome the challenges encountered in this study. We aim to explore new feature extraction strategies and improve the model’s interpretability by investigating contextual relationships between objects within scenes. We will also focus on making the model’s decisions more understandable and exploring multi-modal approaches for better scene understanding. These efforts will enhance the overall effectiveness and adaptability of our scene recognition system.

## Data availability statement

Publicly available datasets were analyzed in this study. This data can be found at: https://www.kaggle.com/datasets/gopalbhattrai/pascal-voc-2012-dataset; https://www.kaggle.com/datasets/sakshaymahna/cityscapes-depth-and-segmentation.

## Author contributions

AA: Data curation, Writing – review & editing. BC: Methodology, Writing – original draft. NM: Investigation, Writing – review & editing. YA: Formal analysis, Writing – review & editing. MA: Resources, Writing – review & editing. HA: Validation, Writing – review & editing. AJ: Supervision, Writing – original draft. HL: Project administration, Writing – review & editing.
